# Management of failed stainless steel implants in the oromaxillofacial region of dogs

**DOI:** 10.3389/fvets.2022.992730

**Published:** 2022-09-23

**Authors:** Janny V. Evenhuis, Frank J. M. Verstraete, Boaz Arzi

**Affiliations:** ^1^Dentistry and Oral Surgery Service, William R. Pritchard Veterinary Medical Teaching Hospital, School of Veterinary Medicine, University of California, Davis, Davis, CA, United States; ^2^Department of Surgical and Radiological Sciences, School of Veterinary Medicine, University of California, Davis, Davis, CA, United States

**Keywords:** plate exposure, osteomyelitis, infection, malocclusion, reconstruction, rh-bone morphogenetic protein-2, titanium, stainless steel

## Abstract

Management of complications of fracture fixation in the oromaxillofacial (OMF) region may present a diagnostic and therapeutic challenge. While titanium and stainless steel implants have been utilized in successful fracture fixation in the OMF region, the use of titanium implants is preferred due to the superior intrinsic properties of titanium. Nonetheless, stainless steel materials are still used due to their availability and familiarity. In the present methods report, we describe our approach to the management of failed stainless steel plates and screws used to treat traumatic injuries in the OMF region. Furthermore, we exemplify our approach with five dogs that exhibited complications of stainless steel implants in the OMF region and their subsequent management. In those cases, all failed implants were removed. Reconstruction with a combination of recombinant human bone morphogenetic protein-2 (rhBMP-2) and titanium implants was utilized in two cases while a mandibulectomy was performed in one case. Three cases required removal of the stainless-steel implant with no additional surgical therapy. We conclude that the success of treatment of failed stainless steel implants depends on the use of advanced imaging findings, appropriate antimicrobial therapy, as well as potentially regenerative reconstructive surgery.

## Introduction

Fractures of the oromaxillofacial (OMF) region present distinct challenges for precise diagnosis and proper fixation techniques given the unique anatomic characteristics of the skull. Maxillofacial fractures result in substantial pain, compromised function, and disfigurement. The most common causes of maxillofacial trauma in the dog include animal bites, vehicular accidents, and blunt force trauma but many result from an unknown etiology (1). The most common locations in the OMF region that are subject to fracture include the maxillary bone and mandibular body (1–3).

Open reduction and internal fixation (ORIF) is one method of fracture fixation in the OMF region and involves the placement of metallic plates and screws to stabilize the fracture fragments. Factors such as fracture morphology, dog anatomy, capacity for healing, and choice of materials play a role in the success or failure of ORIF (4, 5). Hardware for rigid fixation is available in two principal materials: stainless steel and titanium. For fractures of the OMF region, titanium plates and screws are preferred as they offer superior biocompatibility, corrosion resistance, and osteointegration compared with stainless steel (6, 7). The titanium also has an elastic modulus closer to that of bone and is less likely to result in stress shielding of a fracture (8). Furthermore, in the mandible, there is relatively thin soft-tissue coverage over the bone, and erosion of the mucosa overlying stainless steel plates has been documented in mandibular fixation in dogs (9). Results of studies comparing infection resistance between titanium and stainless steel in areas other than the OMF region have been mixed with some studies showing superior infection resistance of titanium and others showing no significant difference between the two materials (10–12). Titanium plates can be manufactured as a lower profile miniplate system that still offers relatively high strength per unit of material weight (8). Nonetheless, it appears that stainless steel plates are still used in OMF fracture fixation in the veterinary setting likely due to the lower cost and wider familiarity of stainless steel materials (7).

Improper use of techniques for OMF fracture fixation may result in fixation failure, non-union, malunion, infection, and implant exposure. Currently, there are no universally accepted human or veterinary protocols for the treatment of exposed hardware in the OMF region. However, in dogs, it is recommended that if the dog is reporting symptoms or displaying signs such as tenderness or pain of the region of injury, fracture instability, fever, or signs of systemic inflammation the infected implant should be removed. Infected tissues should then be debrided, and more effective rigid fixation should be implemented. If the dog shows evidence of appropriate bone healing, remains non-painful, and does not show biochemical evidence of inflammation, then it is not recommended to remove the implant (13, 14). These criteria as reported in human medical studies often prove to be difficult to apply to dogs as complications in people are typically self-reported and follow-up is often limited in dogs.

This methods paper aims to describe the presentation, diagnostic work-up, and management of failed stainless-steel plates and screws that were used for OMF fracture management and exemplify our approach with five clinical cases.

## Materials and methods

### Diagnostic techniques

Dogs that were presented for exposure to a stainless steel plate following an attempt to repair a mandibular fracture underwent pre-anesthetic complete blood count and serum chemistry panel. Under general anesthesia, dogs were then imaged with either conventional computed tomography (CT) (HiSpeed FX/i or LightSpeed16, GE Healthcare, Waukesha, WI) and/or cone-beam computed tomography (CBCT) (NewTom 5G CBCT Scanner, NewTom, Verona, Italy). In selected cases, a pre-contrast and post-contrast CT scan was acquired using intravenous iopamidol (Isovue 370, Bracco Diagnostics, Monroe Township, NJ, USA). All DICOM files from each study were viewed using specialized software (Invivo5, Anatomage, San Jose, CA). Each case was viewed on medical flat-grade flat panel monitors (ASUS PB278Q 27-inch, ASUSTeK Computer Inc., Taipei, Taiwan).

### Surgical technique

Stainless steel orthopedic plate removal was performed with a surgical technique varying with the location of the implant. For dogs with exposed orthopedic plates on the mandible, an extraoral approach was used. The ventral mandible was clipped and aseptically prepared. A skin incision was made over the body of the affected mandible. For bilaterally affected dogs, separate skin incisions were made to approach each mandible. The soft tissues were bluntly and sharply dissected until the ventral aspect of the mandible was visualized. The rostral parts of the digastricus muscle and the facial vein were identified. The orthopedic plate and screws were exposed and the screws were removed using an appropriate screwdriver (i.e., depending on the screw head type). Explanted hardware was submitted for culture and sensitivity testing. Swabs of the surgical site were also submitted for culture and sensitivity testing. The surgical site was flushed copiously with 0.9% sterile saline. Extraoral incisions are closed routinely in two layers of 4-0 poliglecaprone 25 (Monocryl^®^, Ethicon, Somerville, NJ, USA) and one layer of 3-0 nylon (Ethilon^®^, Ethicon, Somerville, NJ, USA). Intraoral incisions were closed in a single layer using 4-0 poliglecaprone 25 (Monocryl^®^, Ethicon, Somerville, NJ, USA). Areas of intraoral hardware exposure were debrided of any diseased soft tissue and closed in a single layer with 4-0 poligecaprone 25 (Monocryl^®^, Ethicon, Somerville, NJ, USA).

Teeth in the region of the implant placement were evaluated radiographically or by CT or CBCT for pathology such as periodontitis, endodontal disease, or iatrogenic trauma. Affected teeth were extracted if indicated.

Cases of non-union fractures were recommended a second reconstructive procedure using a previously published protocol (15). Approximately 4 weeks after failed implant removal, pharyngotomy intubation was performed to facilitate placement of a temporary maxillomandibular fixation (MMF). A 28-G stainless steel wire was placed around the available maxillary and mandibular canine teeth to fixate the maxilla and mandible in normocclusion to act as a temporary MMF. The mandibles were approached through two separate skin incisions using an extraoral approach as previously described. A 2.0-mm locking titanium miniplate or 2.4-mm locking reconstruction plate (Synthes^®^ Maxillofacial, Paoli, PA) was adapted to each fracture site while avoiding tooth root damage. The plate was then secured to the bone with a minimum of 2 (dogs weighing <15 kg) or 3 (dogs weighing >15 kg) locking 3-mm titanium screws in each segment of the fracture. The fracture site was then lavaged with sterile saline. Measurements of the defect were obtained to determine the amount of compression-resistant matrix (CRM - MasterGraft Matrix, Medtronic, Memphis, TN). The CRM was infused with a 50% solution of recombinant human bone morphogenetic protein-2 (rhBMP-2) (Medtronic, Memphis, TN) and soaked for 10 min. The infused CRM was placed at the fracture site and the surrounding soft tissue envelope was closed around the plate and CRM. The wire used in the MMF was removed using wire-cutting forceps.

## Case reports

The record database of the University of California—Davis School of Veterinary Medicine William R. Pritchard Veterinary Medical Teaching Hospital—was searched for stainless steel implant failure cases in dogs presenting from 1st January 2011 to 1st January 2022. To be included for data collection, the orthopedic implants had to be originally placed to treat a traumatic injury in the OMF region and had follow-up surgical treatment. Minimum diagnostic work-up included complete blood count (CBC), serum biochemistry panel, and advanced imaging in the form of CBCT or conventional CT. Data including dog age at the time of presentation, sex, neuter status, breed, and known systemic co-morbidities were recorded. Fracture characteristics such as the mechanism of the initial trauma, presence of periodontal disease before and after implant failure, configuration and location of the fracture, and type of implant used in initial fixation were recorded. Diagnostic testing performed upon the discovery of compromised implant including imaging and culture and sensitivity testing was recorded. Treatments including, whether the implant was surgically removed, types of antibiotics prescribed, type of pain medication prescribed, and any ancillary treatments were also recorded. Additional surgical procedures to further treat the original injury were recorded. Follow-up, as written in the initial record, was noted.

### Case 1

A 13-year-old male castrated Lhasa Apso weighing 8.3 kg was presented for bilateral non-union caudal mandibular fractures. The dog had a history of iatrogenic mandibular fractures during a dental procedure 3 months before the presentation. The right mandibular fracture was repaired with a stainless steel plate and seven stainless steel screws and cerclage wire. The left mandibular fracture was repaired with an orthopedic pin and cerclage wire. One month after the initial repair, the dog demonstrated signs of severe oral pain. A recheck examination revealed a gingival defect with bone exposure on the right mandible in the region of the previous fracture. Culture from the gingival defect revealed a mixed bacterial population with susceptibility to amoxicillin/clavulanic acid. Skull radiographs 29 days later revealed no evidence of bone healing and the dog was referred to University of California–Davis for further management.

On initial presentation, the dog appeared stable but underconditioned with a cloth muzzle in place. An oral examination revealed a mandibular drift and an open bite. Bilateral mucogingival defects with bone exposure were apparent with several visually absent teeth. In addition, an oronasal fistula associated with the region of previously extracted left maxillary second premolar tooth was noted. Preoperative blood work demonstrated a mild neutropenia with a regenerative left shift and a slight hypoglycemia and a mild increase in creatine kinase.

**Figure 1:**
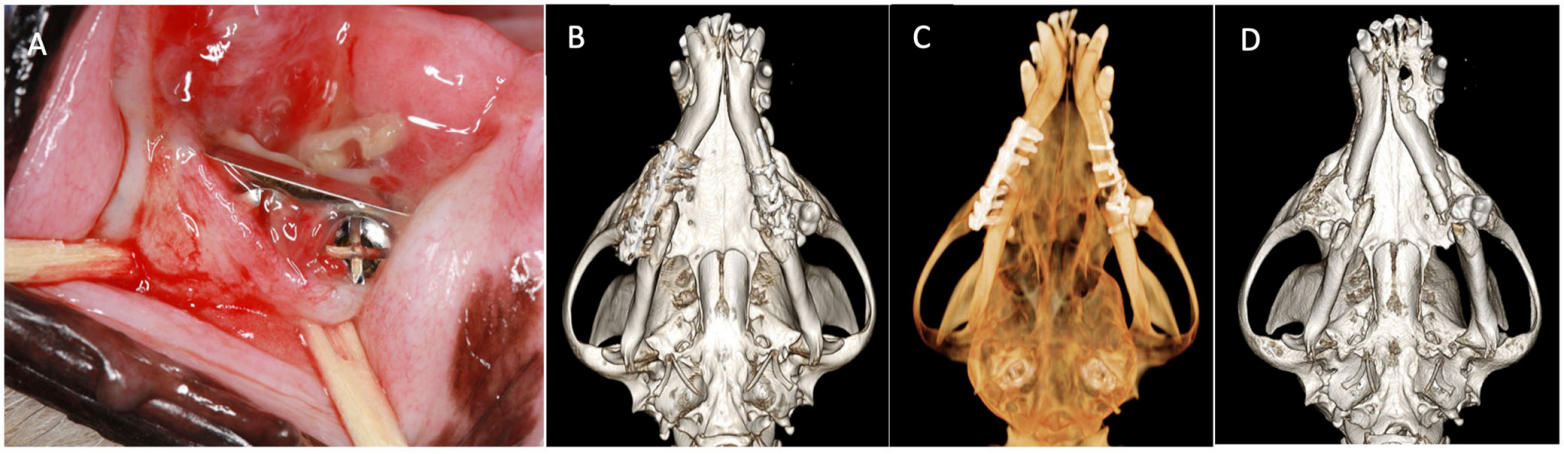
Case 1 images before definitive surgery. **(A)** Intraoral view of right mandibular stainless-steel plate on presentation. **(B)** Three-dimensional bone algorithm CBCT rendering of ventral view of mandibles before removal of a failed implant. **(C)** Three-dimensional tooth algorithm CBCT rendering of ventral view of mandibles before the removal of a failed implant. **(D)** Three-dimensional bone algorithm CBCT rendering of ventral view of mandibles indicating bilateral non-union fractures.

Skull CT with and without contrast revealed bilateral mandibular fractures at the level of the right and left mandibular first molar tooth. The caudal aspect of the stainless steel orthopedic plate on the right side was laterally displaced with noticeable bone loss around the four most caudal screws. The left mandible exhibited a metallic pin traversing both cortices of the caudal left mandible with associated cerclage wires (Figure 1).

The dog underwent removal of all orthopedic implants on both mandibles as described earlier. Extractions of the right mandibular second and third premolar teeth, left mandibular second and fourth premolar teeth, and left and right mandibular first molar teeth were performed due to severe periodontal disease. The culture and sensitivity of the removed implants and bone revealed a mixed bacterial population with some species displaying resistance to several antibiotic classes (Table 1). The dog was discharged from the hospital on amoxicillin/clavulanic acid (22.6 mg/kg two times a day), meloxicam (0.1 mg/kg once a day for 5 days), tramadol (3 mg/kg two times a day for 7–10 days), famotidine (0.6 mg/kg two times a day for 3 days), and chlorhexidine oral rinse after meals and kept in a soft muzzle until definitive repair. Based on the results of the culture and sensitivity tests, the antibiotic therapy was changed to doxycycline (Table 1).

**Table 1:** Summary of antibiotic culture results, susceptibility testing, and initial and final antibiotic therapy.

**Case number**	**Bacterial isolates**	**Resistance**	**Initial antibiotic therapy**	**Final antibiotic therapy**
1	*Enterobacter cloacae*	• Amoxicillin-clavulanate • Ampicillin • Cefazolin Cefpodoxime • Cephalexin	Amoxicillin-clavulanate	Amoxicillin- clavulanate Doxycycline
	*Actinobacter spp*.	Cefazolin		
	*Enterococcus faecium*	• Amoxicillin-clavulanate • Erythromycin • Penicillin • Rifampin		
	*Escherichia coli*			
2	*Pasteurella canis*		Amoxicillin-clavulanate	
3	*Escherichia coli*	• Amoxicillin-clavulanate • Ampicillin • Cefazolin • Cefpodoxime • Cephalexin	• Amoxicillin-clavulanate • Clindamycin	• Clindamycin • Metronidazole^¶^ • Enrofloxacin^¶^ • Amoxicillin-clavulanate^±^
	*Enterococcusfaecalis* ^∧^	• Erythromycin • Rifampin		
4	*Escherichia coli*	Ampicillin	Amoxicillin-clavulanate	Chloramphenicol
	*Enterococcus spp*.	Amoxicillin-clavulanate, Ampicillin, Penicillin, Erythromycin		
	*Staphylococcus spp*.	Amoxicillin-clavulanate, Ampicillin, Cefazolin, Cefovecin, Cefpodoxime, Cephalothin, Imipenem, Oxacillin, Penicillin	Amoxicillin-clavulanate	• Amoxicillin-clavulanate • Marbofloxacin • Doxycyline
	*Pseudomonas aeruginosa*	Amoxicillin clavulanate, Cefazolin		
5	*Neisseria spp*.	Cefazolin	Amoxicillin-clavulanate	Amoxicllin-clavulanate

^¶^Antibiotics added after re-check revealed symptomatic titanium plate exposure.

^∧^Bacteria isolated from a second culture obtained during recheck.

^±^Antibiotic added after results of second culture and sensitivity testing were available.

**Figure 2:**
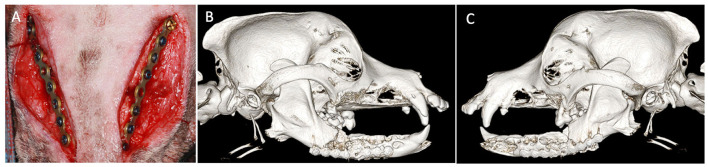
Case 1 intraoperative and postoperative images. **(A)** Intraoperative image showing placement of bilateral titanium miniplates before closure. Three-dimensional bone algorithm CBCT rendering immediately postoperatively of **(B)** right lateral view and **(C)** left lateral view of the skull after placement of titanium miniplates.

**Figure 3:**
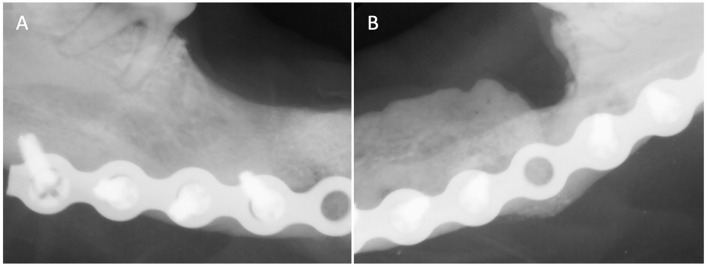
Case 1 follow-up intraoral radiographs of **(A)** right and **(B)** left mandible 3 months post-op showing appropriate bone healing of both fracture sites.

One month later, the dog returned for mandibular reconstruction with titanium locking mini-plates and rhBMP-2 as outlined earlier (Figure 2). The dog was hospitalized for an additional day following surgery and discharged on meloxicam (0.1 mg/kg once a day for 5 days), tramadol (1.6–3.2 mg/kg three times a day for 5–7 days), amoxicillin/clavulanic acid (24.0 mg/kg two times a day for 14 days), and chlorhexidine gluconate oral rinse (two times a day after meals). A follow-up call 7 days after surgery revealed that the dog was recovering clinically well. Further follow-up was performed and intraoral radiographs revealed appropriate healing of the fractures (Figure 3). The owner reported that the dog lived for 4 more years exhibiting a good quality of life and no observed pain or discomfort.

### Case 2

An 8-year-old female spayed miniature poodle weighing 5.1 kg was presented for exposure to a stainless steel orthopedic plate used to repair a left mandibular fracture. The initial fracture repair occurred 3 years before adoption by the current owner and the cause of the initial trauma was unknown. The dog was presented to the referring veterinarian for routine periodontal treatment and examination revealed the increased lateral movement of the mandible with pain. Anesthetized oral examination and radiographs revealed bone loss and a draining tract at the site of the plate and a screw penetrating the lingual mucosa.

**Figure 4:**
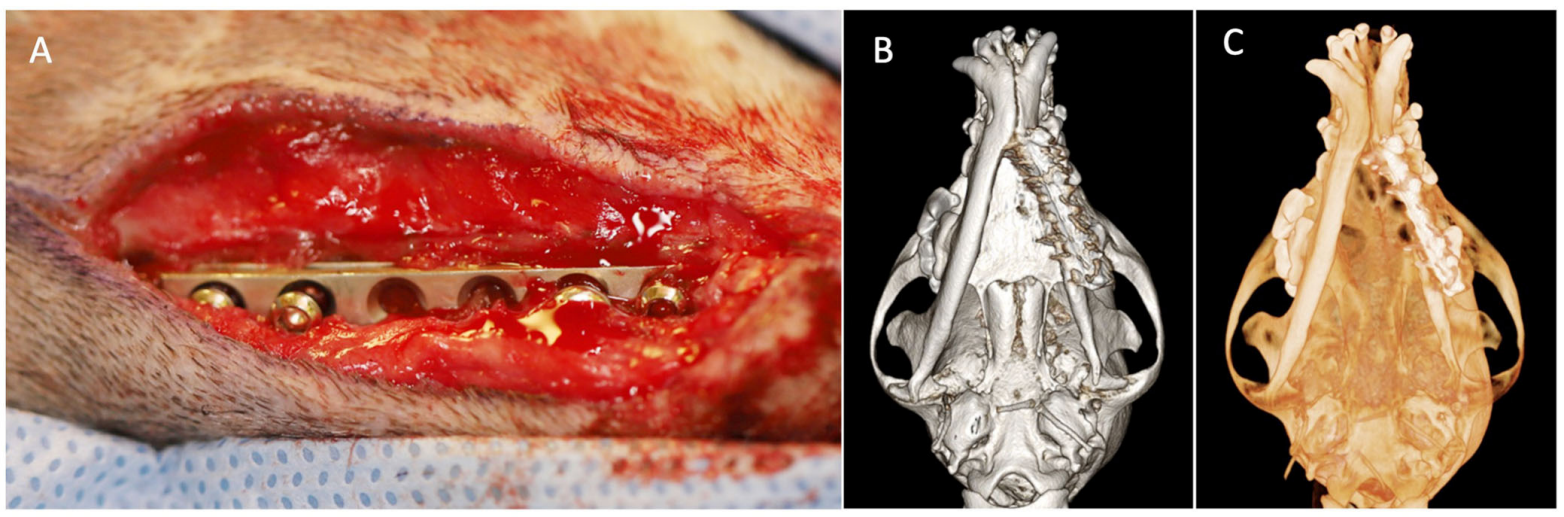
Case 2 images before definitive surgical treatment. **(A)** Intraoperative image showing an approach to left mandible revealing stainless steel plate with loose screws. **(B)** Three-dimensional bone algorithm CBCT rendering of ventral view of mandibles before removal of a failed implant. **(C)** Three-dimensional tooth algorithm CBCT rendering of ventral view of mandibles before removal of a failed implant.

On presentation, the dog appeared cardiovascularly stable. An oral examination revealed mandibular drift to the left with multiple missing teeth. A CBCT and intraoral radiographs revealed an ~15-mm defect extending from the distal root of the left mandibular third premolar tooth to the mesial aspect of the absent left mandibular first molar tooth with evidence of remodeling. A stainless steel plate was noted in the left mandible with one screw apical to the mesial root of the left mandibular third premolar tooth and two screws penetrating the intermandibular space (Figure 4).

The dog underwent removal of the implant as previously described. The left mandibular first, second, and third premolar teeth and left mandibular second molar teeth were extracted. Culture and sensitivity testing of the removed hardware revealed bacterial species showing no resistance to common antibiotics (Table 1). The dog was discharged from the hospital on amoxicillin/clavulanic acid (18.4 mg/kg two times a day for 7 days), buprenorphine (0.018 mg/kg three times a day for 3–5 days), gabapentin (5 mg/kg 2–3 times a day for 5–7 days), and amoxicillin/clavulanic acid (18.4 mg/kg two times a day for 7 days).

A recheck examination and suture removal occurred 2 weeks later and the dog lost ~700 g (13.7% of body weight) since the initial presentation. Mandibular drift to the left was still present and the external surgical incision and extraction sites were healed. A recheck CBCT scan at 6 weeks was recommended.

The owner elected to return the dog for a recheck only 4 months later and a CBCT revealed a slightly larger defect of the left mandible. The dog was doing clinically well and had regained the previous weight. An insufficient amount of bone was present to pursue reconstruction with a titanium locking mini-plate and rhBMP-2. The client elected to perform a left unilateral rostral mandibulectomy extending from the left mandibular canine tooth to the mandibular symphysis. Extraction of additional teeth displaying severe periodontal disease was also performed. The dog was discharged on meloxicam (0.08 mg/kg once a day for 5 days) and gabapentin (5–10 mg/kg 2–3 times a day). Recheck after 2 weeks demonstrated appropriate healing and improved comfort. Mild mandibular drift to the left was persistent as well as mild attrition of the right maxillary canine tooth and right mandibular canine tooth.

### Case 3

A 10-month-old intact male pug weighing 7.2 kg was presented for evaluation of a failed repair of bilateral mandibular fractures. Three weeks before presentation, the dog was attacked by a larger dog and sustained bilateral mandibular fractures. Following CT evaluation, the left mandible was repaired with a stainless steel orthopedic plate. During the initial surgery, a portion of the drill bit broke off and was later located on radiographs within the mandibular canal of the caudal fracture segment. The right mandibular fracture appeared comminuted and rigid fixation was not attempted on the right side. The teeth associated with the fracture were extracted and a circummandibular cerclage wire was placed. On follow-up examinations, the dog appeared painful and the mandible appeared unstable.

Upon presentation, the dog appeared stable but mildly dehydrated. The dog had a previously placed percutaneous endoscopic gastrostomy (PEG) tube in place. An oral examination revealed intraoral left mandibular bone and a stainless-steel implant exposure. A preoperative CBCT showed a left caudal mandibular fracture with an overlying fractured metallic plate as well as a comminuted fracture of the right mandible and the junction of the body and ramus. Additionally, there was a complete fracture through the neck of the left mandibular condylar process and a parasymphyseal fracture extending through the alveolar bone of the left mandibular canine tooth. The dog underwent removal of the left mandibular plate using the approach and techniques described earlier. The previous circummandibular wire appeared to be loose and was replaced. The left mandibular third and fourth premolar teeth and right mandibular first and third molar teeth were extracted due to severe periodontal disease. A postoperative radiograph revealed complete removal of the implants and the absence of the reported drill bit fragment. Postoperative CBCT demonstrated appropriate removal of the bone plate and screws.

The dog was discharged from the hospital on meloxicam (0.09 mg/kg *via* PEG tube once a day for 10 days), gabapentin (5–10 mg/kg *via* PEG tube two times a day for 5–7 days), Tramadol (3.5–7 mg/kg *via* PEG tube two times a day), amoxicillin/clavulanic acid (17.3 mg/kg two times a day for 14 days), and trazodone (3.5–7 mg/kg two times a day). The owners were instructed to keep the dog in a soft muzzle and Elizabethan collar.

At the 1-week recheck, the intraoral surgical site over the left mandible exhibited dehiscence and the area was left for second intention healing. A culture swab of the exposed area was obtained and the dog was then placed on clindamycin (10.4 mg/kg once a day) and chlorhexidine gluconate mouthwash two times a day. Aerobic and anaerobic culture and sensitivity testing revealed a mixed bacterial population including *E. coli* that showed resistance to multiple antimicrobial drugs (Table 1). The dog was continued on clindamycin.

The dog returned approximately 4 weeks after the initial presentation for the repair of the fractures using rigid fixation of the left and right mandibles *via* titanium locking miniplates with rhBMP-2 placement. At the 2-week recheck, the dog appeared to be doing well with no signs of complications. At the 5-week recheck, the dog exhibited infection and dehiscence of the left mandibular surgical site with purulent discharge. Skull radiographs revealed evidence of screw loosening as characterized by lucency around one of the screws and two other screws no longer flush with the plate. The left mandibular titanium plate was removed with the screws and boney sequestra. Unhealthy bone was debrided. Screws and bony sequestra were submitted for culture and sensitivity testing which revealed a multidrug-resistant *E. coli* and also *Enterococcus faecalis* that was susceptible to most antibiotics (Table 1). The dog was prescribed meloxicam (0.1 mg/kg once a day for 10 days), gabapentin (5.5–10 mg/kg two times a day), tramadol (3.3–6.6 mg/kg two times a day), enrofloxacin (9.3 mg/kg once a day for 28 days), and metronidazole (10.2 mg/kg two times a day for 28 days). Following culture and sensitivity results, amoxicillin/clavulanic acid (13.7 mg/kg two times a day for 42 days) was added, and metronidazole was discontinued.

The dog was presented 3 weeks after the removal of the left mandibular titanium implant. Recheck CBCT revealed a widening fracture gap of the left mandible (20 mm compared with 15 mm previously). Clinically, the dog appeared comfortable and functionally was doing well. The right mandible appeared similar to previous imaging with no complete osseous bridging. The amoxicillin/clavulanic acid and enrofloxacin were continued as previously prescribed. Further follow-up was performed elsewhere. Dental radiographs revealed a stable right mandibular fracture repair. Additionally, the dog showed evidence of an atrophic non-union of the left mandible. No additional surgical therapy thus far has been pursued for the mandibular fracture.

### Case 4

A 5-year-old spayed female Boston terrier dog weighing 8.9 kg was presented for mandibular plate exposure. Four months before the presentation, the dog underwent surgical repair of a left caudal mandibular fracture due to a dog bite. Subsequent recheck appointments with referring veterinarian noted malodorous oral discharge and a draining tract in the region of the fracture repair site. Aerobic and anaerobic cultures revealed a mixed bacterial population and the dog was prescribed clindamycin and amoxicillin/clavulanic acid. The dog had no other known systemic diseases at the time of diagnosis.

On presentation to the referral service, the dog appeared cardiovascularly stable. An oral examination revealed an exposed stainless steel plate of the left caudal mandible without palpable mandibular instability. A CBCT showed a non-displaced fracture of the left caudal mandible extending from the left mandibular first molar tooth to the angle of the mandible with incomplete osseous bridging. The metallic plate bridging the fracture gap remained in place with the most rostral screw traversing the mesial root of the left mandibular first molar tooth and the third screw contacting the distal root of the left mandibular first molar tooth. The most caudal screw appeared to be placed below the ventral aspect of the left mandible. Moderate generalized vertical and horizontal bone loss was associated with the remaining dentition.

The dog underwent removal of the left mandibular plate and screws using the methods described earlier. The left mandibular first molar tooth was extracted due to iatrogenic damage from the previously placed screws. Six other teeth were extracted due to severe periodontitis and external inflammatory resorption. Culture and sensitivity testing of the plate and screws revealed a mixed bacterial population displaying resistance to multiple antibiotic classes (Table 1). The dog was prescribed amoxicillin/clavulanic acid (14.2 mg/kg two times a day for 14 days), carprofen (1.4 mg/kg two times a day for 5–7 days), and gabapentin (11.3 mg/kg two times a day).

The dog was presented at the 1-week recheck and appeared clinically normal with appropriately healing surgical sites. After receiving the results of the culture and sensitivity testing, the amoxicillin/clavulanic acid was discontinued and chloramphenicol (28.4 mg/kg PO three times a day for 14 days) was started. The dog exhibited appropriate occlusion for the breed, good healing of the surgical site, and continued to do well at the 2-week recheck.

### Case 5

A 9-year-old spayed female American Eskimo dog weighing 6.2 kg was presented for left mandibular stainless steel plate exposure. Approximately 1.5 years earlier, the dog underwent surgical repair of a left mandibular fracture due to a dog bite. During a routine periodontal treatment with the referring veterinarian, the plate was noted to be exposed. The client had not noticed any overt pain but the dog seemed to be more careful when eating food.

**Figure 5:**
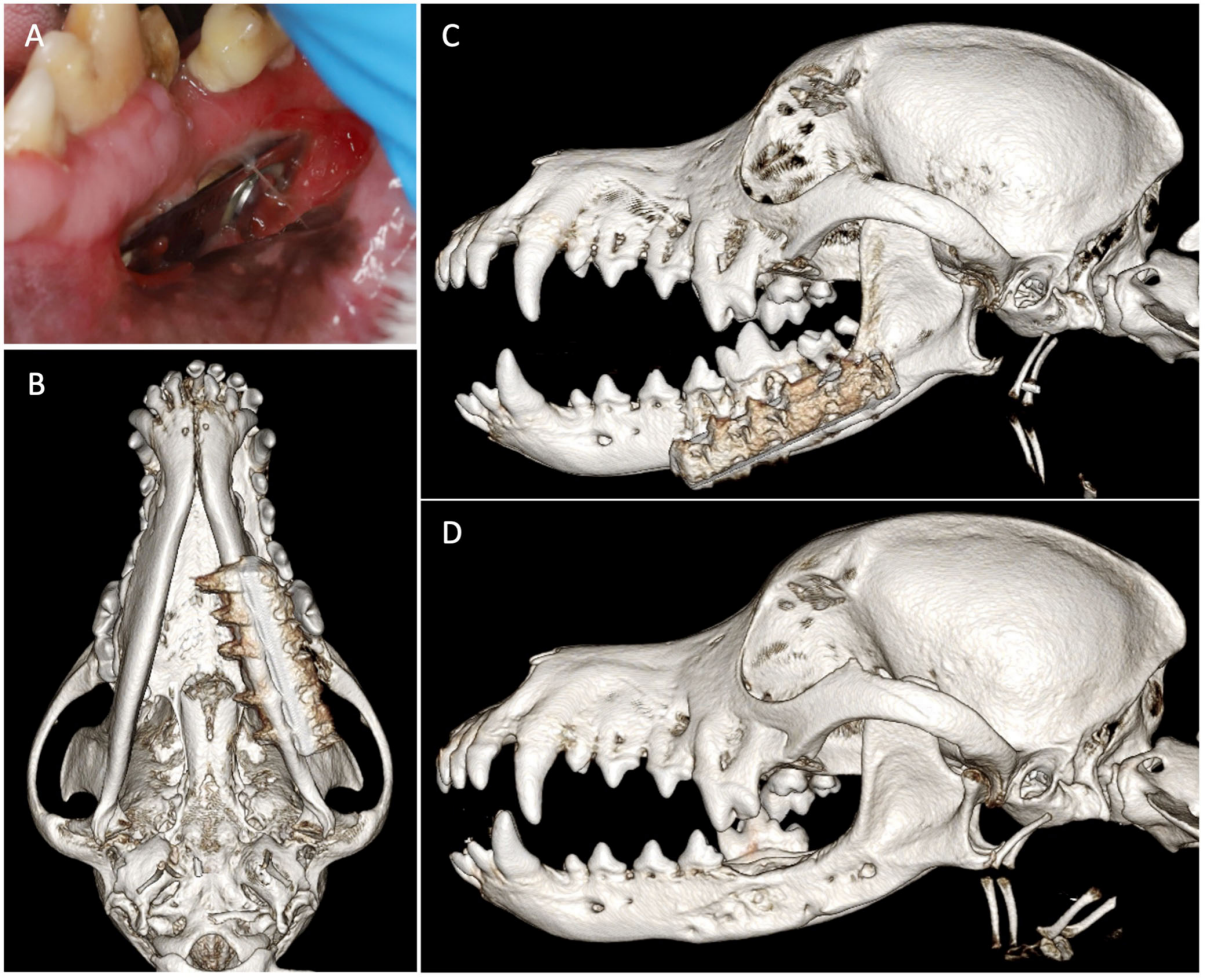
Case 5 images before and after implant removal. **(A)** Preoperative image showing intraoral left mandibular stainless steel plate exposure. Three-dimensional bone algorithm CBCT rendering of **(B)** ventral view and **(C)** left lateral view of skull before removal of the exposed implant. **(D)** Three-dimensional bone algorithm CBCT rendering of the left lateral view of the skull at 6 weeks after implant removal.

The dog was cardiovascularly stable on a general physical exam. An oral examination revealed a stainless steel plate visible intraorally along the lateral aspect of the left mandible. The mandible felt stable on palpation. CBCT scan revealed that the plate was present along the lateral aspect of the left mandible and horizontal and vertical bone loss was associated with the left mandibular first, second, and third molar teeth. The margins of the previous fracture were ill-defined with evidence of osseous bridging (Figures 5A–C). A periapical lucency was associated with the mesial root of the left mandibular first molar tooth.

The dog underwent removal of the left mandibular stainless-steel plate using the methods described above. The left mandibular first, second, and third molar teeth were extracted due to periapical lucency and severe attachment loss. A culture swab of the stainless-steel plate submitted for aerobic and anaerobic culture and sensitivity testing revealed a mixed bacterial population with *Neisseria spp*. showing resistance to cefazolin (Table 1). The dog was prescribed carprofen (2.0 mg/kg two times a day for 7 days), gabapentin (8.0 mg/kg two times a day), and amoxicillin/clavulanic acid (15.1 mg/kg two times a day for 42 days). The dog was also placed in a soft nylon muzzle for 2 weeks postoperatively.

At the 2-week recheck, the dog appeared to be eating and drinking normally and comfortably. The surgical sites appeared appropriately healed and the muzzle was removed. At the 6-week recheck, the dog underwent a recheck CBCT (Figure 5D). The dog exhibited mild drifting of the mandible to the right, without causing soft tissue trauma, but the mandible was palpably stable. The previous fracture site exhibited further remodeling including filling of the previous screw holes and emptying alveoli with new bone. No further treatment was recommended.

## Discussion

In this methods paper, we describe our approach to the management of failed stainless steel implants in the OMF region and exemplify with reports on our experience with 5 dogs. We demonstrated that exposed stainless steel implants in the OMF region require a step-wise approach that begins with clinical evaluation, advanced diagnostic imaging, and surgical management to restore dog comfort and function. Affected dogs typically present with evidence of oral pain and infection, delayed or lack of healing, and/or inability to prehend food. Removal of exposed and infected implants is required as well as obtaining diagnostic samples for culture and sensitivity to inform precise antibiotic therapy. For chronic non-union fractures, reconstruction using a titanium locking miniplate together with rhBMP-2 infused on CRM may be considered. If there is evidence of appropriate bone healing with no deficits in the ability of the dog to eat and drink unassisted, removal of the exposed implant and antibiotic therapy may suffice.

While it is difficult to assert the cause of stainless steel implant failure in these cases, proper surgical technique and choice of materials may prevent undue consequences. Previous studies in human and veterinary medicine have explored the use of stainless steel implants in the OMF region. While both materials have been successful in the treatment of fractures in the OMF region, titanium plates were demonstrated to be more corrosion resistant (16). Additionally, in one study, two-third of dogs exhibited erosion of intraoral soft tissue covering a stainless steel plate and 61% of stainless steel screws caused trauma to adjacent teeth with the use of stainless steel ORIF systems following experimental osteotomy (9). Similar complications such as implant exposure and trauma to adjacent teeth have been reported with the utilization of titanium maxillofacial plates and rh-BMP following oral mass resection or facial trauma. In some cases, exposed titanium implants required removal and in other cases removal of the implant was avoided (17, 18). One case report observed implant exposure, sequestrum formation, implant breakage, and impaction of a permanent tooth when titanium maxillofacial plates were used to treat mandibular fractures in a juvenile dog (19). In Case 3, the removal of a titanium miniplate was performed following the dehiscence of overlying soft tissue. It is unknown how material choice contributed implant failure in Case 3 but mechanisms of failure are likely multifactorial and may include implant choice, surgical technique, aftercare, patient biomechanics, and the anatomic changes from the previous surgery. Therefore, titanium implants used in the maxillofacial region may be subject to similar complications to stainless steel materials although rates of complications have not been reported in veterinary species at this juncture. Nonetheless, titanium implants offer superior biocompatibility and osteointegration and are manufactured in low-profile systems that are ideal for the OMF region (6, 7, 16).

Titanium implants have a rough microsurface that supports direct osteointegration when compared with the electropolished stainless steel (EPSS) that is most commonly used in orthopedic implants. Furthermore, titanium owes its superior corrosion resistance to the formation of a thicker metal oxide layer (5–6 nm) compared with EPSS (2–3 nm). Additionally, the equilibrium of a titanium oxide layer is more quickly re-established when it is disrupted compared with a chromium oxide layer. The titanium also has a lower modulus of elasticity when compared with EPSS and its modulus of elasticity is closer to that of bone (7). While a lower modulus of elasticity may not be advantageous in weight-bearing bones, our experience demonstrates that it is acceptable in the maxillofacial region where structures are subject to comparatively lighter loads and may allow for enough load bearing of native bone to facilitate normal bone healing. We can comfortably say that in this case series, all of the plates used for initial fracture fixation were stainless steel plates from various manufacturers, and it is possible that the plate macrostructure contributed to impaired healing (20, 21).

The placement location of the implant and screws are also an important factor in the success of fracture fixation. For example, two out of five cases presented here demonstrated trauma to adjacent teeth due to screw placement. Trauma to the adjacent tooth roots may result in pulpitis and pulp necrosis, which can progress to the formation of periapical pathology and periodontal disease as noted in the above cases. Ultimately, this may compromise rigid fixation, healing, and comfort. Prospective studies comparing outcomes of titanium vs. stainless steel materials are required to truly examine how differences in materials translate to differences in outcomes. Although no formal studies have been conducted in veterinary medicine to compare the use of stainless steel and titanium materials, titanium is the preferred metal for the treatment of maxillofacial injuries in human patients due to its biocompatibility, osteointegration, and biomechanical properties (22, 23). The mechanism of implant failure in these cases is likely multifactorial and may represent the culmination of choice of surgical materials, surgical technique, fracture morphology, and aftercare (5).

Dogs in this report underwent variable surgical therapy, ranging from removal of the exposed implant with no additional surgical therapy to the reconstruction of non-union fractures with a locking titanium miniplate combined with rhBMP-2 infused on a CRM. Two dogs required removal of the implant and extraction of diseased teeth as the main surgical intervention. No additional fixation was required as enough bone healing had occurred to stabilize the fracture fragments. However, it is possible that if inflammation was not treated and bone loss progressed, bone healing would have eventually been compromised. The decision to proceed with the usage of a titanium plate with or without rhBMP-2 is dependent on subsequent progress examination and re-imaging. If the patient exhibits evidence of fracture fragment stability and pain-free function, additional surgical procedures may not be necessary. One dog underwent a unilateral rostral mandibulectomy in response to continued osteomyelitis, bone resorption, and mandibular non-union. For this dog, insufficient bone remained to place a titanium plate and rhBMP-2. The mandibulectomy surgery served to debride infected bone associated with the previous fixation.

Reconstruction techniques using rhBMP-2 as used in Case 1 and Case 3 are indicated in dogs that have a chronic (>6 weeks) critical size defect (i.e., 15 mm without intact periosteum or a bone defect that is unlikely to heal during the patient lifetime) that is showing no evidence of healing and has associated malocclusion (15). For mandibular reconstruction using the reported technique, dogs should have enough bone remaining to allow the placement of a minimum of two locking screws on each side of the bone fragment (15, 17). Regenerative techniques as described in the current manuscript are not ideal in dogs that have a stable bone callus with incomplete healing or in dogs with concern of pathologic fracture due to an underlying neoplastic process. The need for repeated anesthetic episodes in the context of dog co-morbidities, owner compliance, and the likelihood of follow-up should also be considered during case selection.

Removal of the exposed and failed implants is the most essential step in allowing the bone and soft tissue to heal. In addition, obtaining culture and sensitivity in conjunction with implant removal is recommended as was performed in all cases in this report. All but one case exhibited bacterial species with resistance to at least one antibiotic class which underscores the importance of culture and sensitivity testing. However, culture and sensitivity testing should be evaluated critically in the context of the dog's response to therapy as an escalation of therapy may not be warranted if the dog is responding well. Ultimately, the antibiotic choice should be efficacious, targeted, and adhere to the principles of antimicrobial stewardship so as not to precipitate further antibiotic resistance (24). Local therapy including chlorhexidine gluconate rinse may also be a sensible addition to a postoperative regimen. However, resistance to chlorhexidine local therapy in the oral cavity is a rising concern in human and veterinary dentistry (25). In the above cases, the influence of culture and sensitivity testing and subsequent antibiotic choice on the outcome is difficult to assess without controlled studies. Nonetheless, addressing the underlying mechanisms of abnormal bacterial colonization (i.e., the non-healing injury and/or exposed hardware) are paramount as infection will not resolve with systemic antibiotic administration alone.

In this case series, we outline the medical and surgical management of exposed stainless steel implants and demonstrate our experience in five cases. Stainless steel implant complications can present in a variety of forms and thus treatment may vary greatly. Removal of the compromised implant is recommended as the material properties of stainless steel and the configuration of the implant likely contributed to complications (5). Reconstruction of non-healing defects with rhBMP-2 may be pursued, but case selection must be precise to increase the chance of success. Finally, antibiotic choice and follow-up including repeat CT are recommended to ensure proper healing and return to function.

## Data availability statement

The raw data supporting the conclusions of this article will be made available by the authors, without undue reservation.

## Ethics statement

Ethical review and approval was not required for the animal study because it is a retrospective case series. Written informed consent was obtained from the owners for the participation of their animals in this study.

## Author contributions

JE: study concept and design, data collection, data analysis and interpretation, and manuscript writing. BA: study concept and design, provision of study material or cases, data analysis and interpretation, manuscript writing, and review of the manuscript for important intellectual input. FV: study concept and design, provision of study material, and review of the manuscript for important intellectual input. All authors contributed to the article and approved the submitted version.

## Conflict of interest

The authors declare that the research was conducted in the absence of any commercial or financial relationships that could be construed as a potential conflict of interest.

## Publisher's note

All claims expressed in this article are solely those of the authors and do not necessarily represent those of their affiliated organizations, or those of the publisher, the editors and the reviewers. Any product that may be evaluated in this article, or claim that may be made by its manufacturer, is not guaranteed or endorsed by the publisher.

## References

[1] De Paolo MH Arzi B Pollard RE Kass PH Verstraete FJM. Craniomaxillofacial trauma in dogs—Part I: fracture location, morphology and etiology. Front Vet Sci. (2020) 7:241. 10.3389/fvets.2020.00241 32411743 PMC7199291

[2] Lopes FM Gioso MA Ferro DG Leon-Roman MA Venturini MAFA Correa HL. Oral fractures in dogs of Brazil — A retrospective study. J Vet Dent. (2005) 22:86–90. 10.1177/089875640502200202 16149386

[3] Umphlet RC Johnson AL. Mandibular fractures in the dog a retrospective study of 157 cases. Vet Surg. (1990) 19:272–5. 10.1111/j.1532-950X.1990.tb01184.x 2382396

[4] Boudrieau RJ Arzi B. 33 - Maxillofacial fracture repair using plates and screws. In: Verstraete FJM, Lommer MJ, Arzi B, editors. Oral and Maxillofacial Surgery in Dogs and Cats. 2nd ed. Philadelphia, PA: Elsevier (2020). p. 319–38.

[5] Bar-Am Y Marretta SM. 35 - Maxillofacial fracture complications. In: Verstraete FJM, Lommer MJ, Arzi B, editors. Oral and Maxillofacial Surgery in Dogs and Cats. 2nd ed. Philadelphia, PA: Elsevier (2020). p. 351–60.

[6] Langford RJ Frame JW. Surface analysis of titanium maxillofacial plates and screws retrieved from patients. Int J Oral Maxillofac Surg. (2002) 31:511–8. 10.1054/ijom.2002.0283 12418567

[7] Hayes J Richards R. The use of titanium and stainless steel in fracture fixation. Expert Rev Med Devices. (2010) 7:843–53. 10.1586/erd.10.53 21050093

[8] De Zeeuw L Scheunemann O. Materials and Instrumentation. Atlas of Craniomaxillofacial Osteosynthesis. 2nd ed. Stuttgart: Thieme Verlag (2009).

[9] Verstraete FJM Ligthelm AJ. Dental trauma caused by screws in internal fixation of mandibular osteotomies in the dog. Vet Comp Orthop Traumatol. (1992) 5:104–8. 10.1055/s-0038-1633078

[10] Arens S Schlegel U Printzen G Ziegler W Perren S Hansis M. Influence of materials for fixation implants on local infection. Bone Joint J. (1996) 78:647–51. 10.1302/0301-620X.78B4.0780647 8682836

[11] Moriarty TF Debefve L Boure L Campoccia D Schlegel U Richards RG. Influence of material and microtopography on the development of local infection *in vivo*: experimental investigation in rabbits. Int J Artif Organs. (2009) 32:663–70. 10.1177/039139880903200916 19882548

[12] Metsemakers WJ Schmid T Zeiter S Ernst M Keller I Cosmelli N . Titanium and steel fracture fixation plates with different surface topographies: Influence on infection rate in a rabbit fracture model. Injury. (2016) 47:633–9. 10.1016/j.injury.2016.01.011 26830128

[13] Matthew IR Frame JW. Policy of consultant oral and maxillofacial surgeons towards removal of miniplate components after jaw fracture fixation: pilot study. Br J Oral Maxillofac Surg. (1999) 37:110–2. 10.1054/bjom.1997.0084 10371312

[14] Cahill TJ Gandhi R Allori AC Marcus JR Powers D Erdmann D . Hardware removal in craniomaxillofacial trauma. Ann Plast Surg. (2015) 75:572–8. 10.1097/SAP.0000000000000194 25393499 PMC4888926

[15] Verstraete FJM Arzi B Huey DJ Cissell DD Athanasiou KA. Regenerating mandibular bone using rhBMP-2: Part 2—Treatment of chronic, defect non-union fractures. Vet Surg. (2015) 44:410–6. 10.1111/j.1532-950X.2014.12122.x 24410723 PMC4451182

[16] Torgersen S Gjerdet NR. Retrieval study of stainless steel and titanium miniplates and screws used in maxillofacial surgery. J Mater Sci Mater Med. (1994) 5:256–62. 10.1007/BF00122394

[17] Arzi B Verstraete FJM Huey DJ Cissell DD Athanasiou KA. Regenerating mandibular bone using rhBMP-2: Part 1—Immediate reconstruction of segmental mandibulectomies. Vet Surg. (2015) 44:403–9. 10.1111/j.1532-950X.2014.12123.x 24410740 PMC4451165

[18] Boudrieau RJ. Initial experience with rhBMP-2 delivered in a compressive resistant matrix for mandibular reconstruction in 5 dogs. Vet Surg. (2015) 44:443–58. 10.1111/j.1532-950X.2014.12171.x 24617340

[19] Basuki W Rawlinson JE Palmer RH. Repair of bilateral comminuted mandibular fractures in a 12-week-old puppy using locking and nonlocking maxillofacial reconstruction plates. J Vet Dent. (2018) 35:258–67. 10.1177/0898756418812818

[20] Jain R Podworny N Hupel TM Weinberg J Schemitsch EH. Influence of plate design on cortical bone perfusion and fracture healing in canine segmental tibial fractures. J Orthop Trauma. (1999) 13:178–86. 10.1097/00005131-199903000-00005 10206249

[21] Johnston SA von Pfeil DJF Dejardin LM Roe SC Weh JM. Internal fracture fixation. In: Johnston SA, Tobias KM, editors. Veterinary Surgery: Small Animal. 2nd ed. St. Louis, Missouri: Elsevier (2018). p. 654–90.

[22] Pacifici L De Angelis F Orefici A Cielo A. Metals used in maxillofacial surgery. Oral Implantol. (2017) 14:107–11. 10.11138/orl/2016.9.1S.107 28280540 PMC5333745

[23] Hupp J Ellis E Tucker M (editors). Contemporary Oral and Maxillofacial Surgery. 7th ed. St Louis, MI: Elsevier (2019).

[24] Lloyd DH Page SW. Antimicrobial stewardship in veterinary medicine. Microbiol Spectr. (2018) 6:3–6. 10.1128/microbiolspec.ARBA-0023-2017 29916349 PMC11633576

[25] Cieplik F Jakubovics NS Buchalla W Maisch T Hellwig E Al-Ahmad A. Resistance toward chlorhexidine in oral bacteria – is there cause for concern? Front Microbsiol. (2019)10: 587. 10.3389/fmicb.2019.00587 30967854 PMC6439480

